# Stimulation-related modifications of evolving functional brain networks in unresponsive wakefulness

**DOI:** 10.1038/s41598-022-15803-5

**Published:** 2022-07-08

**Authors:** Christoph Helmstaedter, Thorsten Rings, Lara Buscher, Benedikt Janssen, Sara Alaeddin, Vanessa Krause, Stefan Knecht, Klaus Lehnertz

**Affiliations:** 1 St. Mauritius Therapieklinik GmbH, Strümper Str. 111, 40670 Meerbusch, Germany; 2 Department of Epileptology, University of Bonn Medical Centre, Venusberg Campus 1, 53127 Bonn, Germany; 3 Helmholtz Institute for Radiation and Nuclear Physics, University of Bonn, Nussallee 14-16, 53115 Bonn, Germany; 4 Interdisciplinary Center for Complex Systems, University of Bonn, Brühler Str. 7, 53175 Bonn, Germany

**Keywords:** Psychology, Medical research, Neurology, Physics

## Abstract

Recent advances in neurophysiological brain network analysis have demonstrated novel potential for diagnosis and prognosis of disorders of consciousness. While most progress has been achieved on the population-sample level, time-economic and easy-to-apply personalized solutions are missing. This prospective controlled study combined EEG recordings, basal stimulation, and daily behavioral assessment as applied routinely during complex early rehabilitation treatment. We investigated global characteristics of EEG-derived evolving functional brain networks during the repeated (3–6 weeks apart) evaluation of brain dynamics at rest as well as during and after multisensory stimulation in ten patients who were diagnosed with an unresponsive wakefulness syndrome (UWS). The age-corrected average clustering coefficient C* allowed to discriminate between individual patients at first (three patients) and second assessment (all patients). Clinically, only two patients changed from UWS to minimally conscious state. Of note, most patients presented with significant changes of C* due to stimulations, along with treatment, and with an increasing temporal distance to injury. These changes tended towards the levels of nine healthy controls. Our approach allowed to monitor both, short-term effects of individual therapy sessions and possibly long-term recovery. Future studies will need to assess its full potential for disease monitoring and control of individualized treatment decisions.

## Introduction

Acute brain damage can lead to severe disorders of consciousness (DOC). The changing age structure of the population and improved medical care for acute neurological diseases of the central nervous system (CNS) cause an increasing number of survivors with an unresponsive wakefulness syndrome (UWS, synonyms: vegetative state, apallic syndrome) or with a minimally conscious state syndrome (MCS) in an intensive care setting. A systematic review from 2014 on studies of the prevalence of UWS shows a wide range from 0.2/100,000 to 6.1/100,000 inhabitants^1^. In Germany, between 1500 and 5000 patients are in such a condition^2^. The reported prevalence values, however, vary considerable, which has been attributed to factors like the quality and availability of emergency and intensive care services as well as different cultural, political, professional, and judicial attitudes towards end-of-life decisions in different clinical settings^3, 4^.

Patients with chronically impaired consciousness are among the most vulnerable patient groups. They are exposed to the help and interests of others. Intensive care can recover functions of phylogenetically and ontogenetically older life-sustaining brain structures, but higher cortical, cognition- and consciousness-supporting structures are damaged with an often uncertain outcome. The capability of observing consciousness would allow therapies to be better adapted to the individual needs of each patient. Moreover, it would enable to decide how long life support should be maintained^5, 6^. Other relevant issues connected to this are pain (“does the patient suffer?”), brain death, and organ donation^7, 8^.

Electroencephalography (EEG) provides a simple and reliable way to access the brain’s activity in a state of unresponsiveness^9, 10^. In this regard, great hopes are currently being placed on new developments that indicate the presence and the degree of brain functionality and the prognosis of the recovery of consciousness using functional-imaging-based (e.g. functional magnetic resonance imaging, fMRI) or EEG-based network analyses^11–21^. A meta-study on the persistent vegetative state and minimal state of consciousness from 2015 examined 20 studies with a total of 906 patients and compared different methods of assessment^2^. The overall conclusion is that positron emission tomography (PET) and quantitative EEG examinations in particular provide complimentary information in different states of consciousness. Resting-state EEG and fMRI are the state-of-the-art techniques with a huge potential for predicting the recovery of coma patients. Apart from relying on resting-state conditions, electrophysiological and imaging studies have made use of different stimulation conditions including transcranial magnetic stimulation or electric stimulation to assess and to predict recovery of consciousness^22, 23^. However, as pointed out in a recent systematic review on the role of electrophysiology in DOC, complex recording protocols, a confusing variety of EEG measures, and complicated analysis algorithms yet prevent a broader systematic application^24^.

From a methodological and particularly from a pragmatic point of view, the following questions should be asked of any method for the monitoring of brain activity changes associated with consciousness and for the prognosis of DOC:

- Which paradigms and analysis methods are valid and reliable (repeatable) in the individual patient?
- Is the method suitable for different types of traumatic brain injuries and for the conditions of acute, subacute and chronic damage?
- How objective and easy is the data evaluation?
- What are the costs, the technical and logistical efforts, and the availability on site?

With a view to answering these questions in the future, we investigated patients who—from a clinical point of view—were indistinguishable with the diagnosis of an unresponsive wakefulness syndrome (UWS). Specifically, we tracked—in a time-resolved manner—important global characteristics (average clustering coefficient and average shortest path length) of the patients' EEG-derived evolving functional brain networks during a repeated evaluation of brain activity at rest as well as during and after therapeutic basal stimulation. The average clustering coefficient *C* characterises a network’s functional segregation; the higher *C*, the more segregated is the network. The average shortest path length *L* characterises a network’s functional integration; the shorter *L*, the more integrated is the network.

Our proof-of-principle study aims at a first validation of these global network characteristics for a repeated assessment and automated evaluation on the level of the individual patient. We investigated repeated resting-state recordings and recordings during multisensory stimulation in a block design. At this initial stage of the investigation, we did not yet differentiate between individual types of sensory stimulation (auditory, visual, tactile) within the stimulation block. Thus, the study design differs from previous studies that focused solely on resting-state and/or individual stimulation types^22, 24^, as well as from studies, which evaluated functional brain networks on a population-sample level^11, 21, 25^. However, our study design raises the idea that a repeated assessment might reveal additional information^26^. In particular, we addressed the following three research questions: (I) do global network characteristics differ in patients with clinically indiscriminate states of unresponsive wakefulness/UWS (improved patient classification)? (II) Do global network characteristics indicate functional recovery and success of continuous early rehabilitation complex treatment (long-term treatment effects)? And (III), do global network characteristics indicate the brain’s response to single basal multisensory therapeutic interventions (short-term treatment effects)?

## Results

We monitored a group of ten patients (“Methods”, Table 3) diagnosed with an unresponsive wakefulness syndrome who had been submitted for early rehabilitation to the St. Mauritius neuro-rehabilitation center in Meerbusch, Germany during the period 2019 to 2020. The patients’ Coma Recovery Scale (CRS-R; “Methods”) scores ranged between 2 and 8 of 23 possible points. In addition, we investigated nine healthy controls (five females; age 26–60 years).

**Figure 1:**
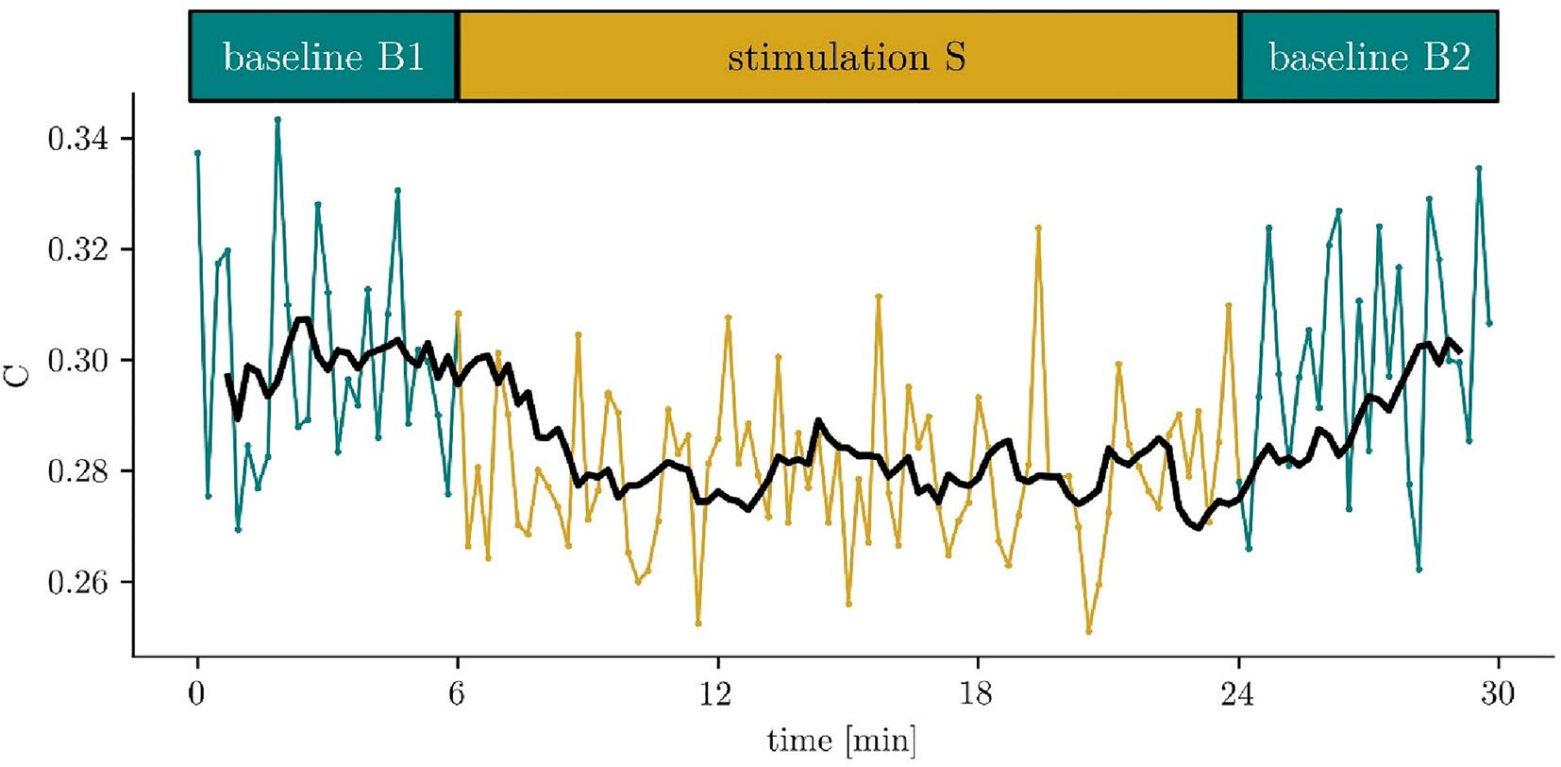
Top: examination schedule comprising two baseline conditions (6 min. each) prior to and after a multisensory stimulation block (18 min.) probing the somatosensory, auditory, and visual modalities. Bottom: exemplary temporal evolution of the clustering coefficient along the examination schedule. A 7-point sliding average is indicated by the black line.

In addition to the daily rehabilitation complex treatment (“Methods”), all patients were evaluated twice with EEG recordings (“Methods”) within a time frame of 3–6 weeks. Along with each assessment, the CRS-R was documented. The two assessments (A1 and A2) lasted about 30 min each and so did the EEG recording. Each assessment followed the same schedule (Fig. 1): a 6-min pre-stimulation baseline (B1), followed by three 3-min stimulation blocks (stimulation S), separated by two 3-min rest conditions, followed by a 6-min post-stimulation baseline (B2). Stimulation blocks comprised stimulations in three modalities: somatosensory, auditory, and visual. For each modality, different stimuli were used in an alternating sequence (cold, massage, brushing/rubbing; clapping hands, buzzing tone, speech; flashing light, still images, moving object). We took care that all patients had their eyes open during the assessments. All patients were moved into a supine position and exposed to each type of stimulation for one minute (30 s for each side of the body), always starting at the left side.

Healthy controls were evaluated in single sessions and were instructed to let their thoughts run freely during the whole assessment. Controls were awake and had their eyes open, as this condition would be the ultimate therapeutic aim to reach in patients with DOC.

### Global network characteristics allow additional patient classification

**Figure 2:**
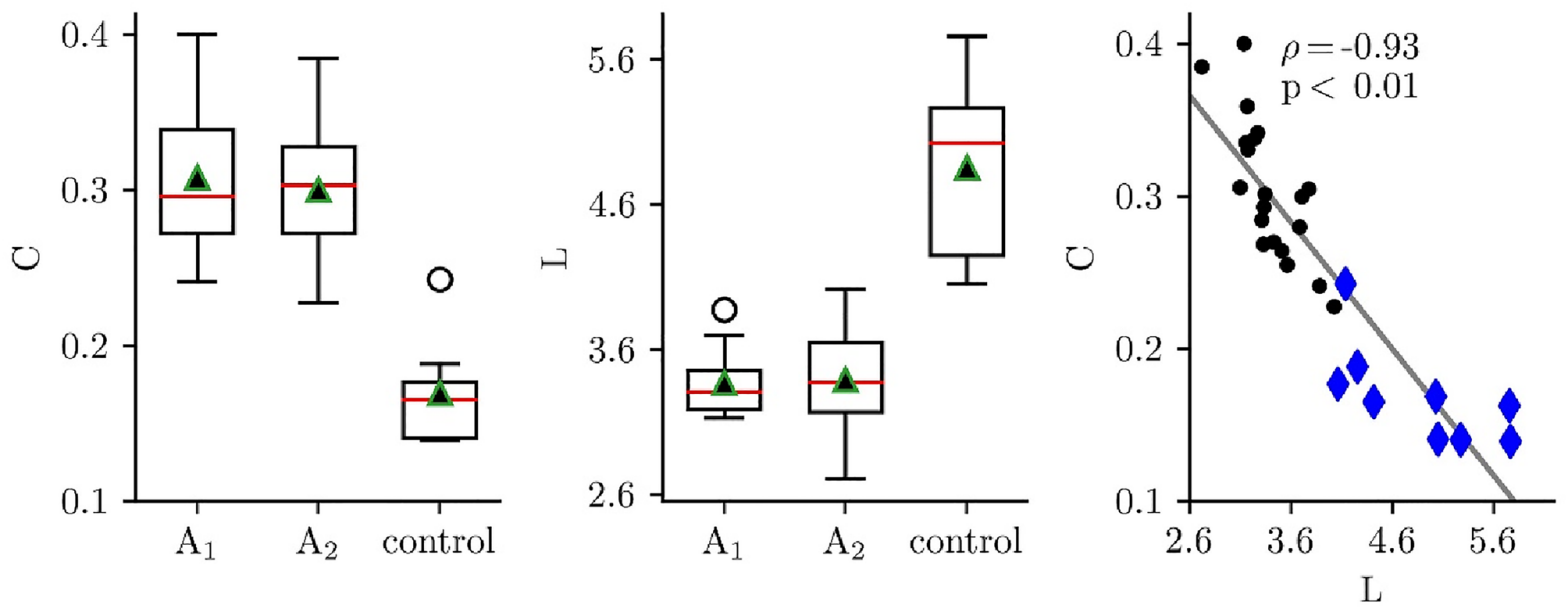
Sample distributions of average clustering coefficient *C* (left) and average shortest path length *L* (middle) of evolving functional brain networks from patients during period B1 at first (**A1**) and second assessment (**A2**) and from healthy controls. Bottom and top of a box are the first and third quartiles, and the red band and the black triangle are the median and the mean of the distribution, respectively. The ends of the whiskers represent the interquartile range of the data. Outliers are marked by an o sign. Right: Scatterplot of average clustering coefficients *C* and average shortest path lengths *L* during period B1 from patients (both assessments; black dots) and healthy controls (single assessment; blue diamonds). Linear regression (all subjects) is represented with a solid black line (patients: Spearman’s ρ = − 0.80, *p* < 0.01; healthy controls: ρ = − 0.88, *p* < 0.01).

We first examined whether the global characteristics of evolving functional brain networks (“Methods”) allow for an additional patient classification. In Fig. 2, we present the patients’ distributions of average clustering coefficients *C* and average shortest path lengths *L* of evolving functional brain networks during period B1 at first and second assessment (A1 and A2) together with the distributions of the healthy controls. There was a large interindividual variability despite the fact that all patients were clinically classified as UWS. As it can be taken form Fig. 2, both global network characteristics differentiated the patients from healthy controls. However, a more detailed investigation revealed that the characteristics demonstrated a highly significant inverse relationship (Spearman’s ρ = − 0.93; *p* < 0.01): the lower *C* the higher *L*. We therefore restricted all subsequent analyses to the average clustering coefficient.

**Figure 3:**
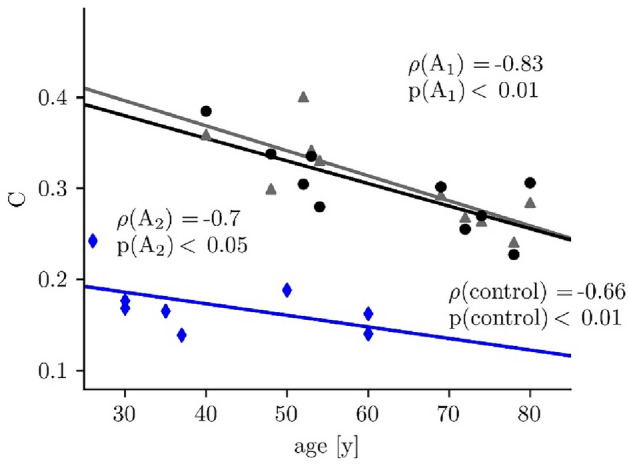
Scatterplot of average clustering coefficients *C* (all conditions: B1, S, B2) and age from patients at both assessments (**A1**: grey triangle; **A2**: black dot) and healthy controls (blue). Solid lines represent linear regressions (Spearman’s correlation coefficient ρ).

Searching for the source of the large interindividual variability of the average clustering coefficient, we observed this network characteristic to be strongly influenced by age: the higher the age the lower the average clustering coefficient C (Fig. 3). This was the case for first assessment (A1: Spearman’s ρ = − 0.83, p < 0.01) and could be confirmed for the second assessment (A2: Spearman’s ρ = − 0.7, p < 0.05). The age-effect was also observed in the healthy control group (Spearman’s ρ = − 0.66, p < 0.01).

In order to avoid a possible confounding effect on subsequent analysis steps, we proceeded with age-corrected average clustering coefficients (C* = *C* – age [y] *s* [1/y]) to investigate whether this network characteristic allows for an additional patient classification (patients: *s* = − 0.0025; healthy controls: *s* = − 0.0013). The clear cut differences seen for the average clustering coefficients between patients and controls (Fig. 2) remained highly significant after age correction (Mann–Whitney *U* = 2, *p* < 0.0001).

**Figure 4:**
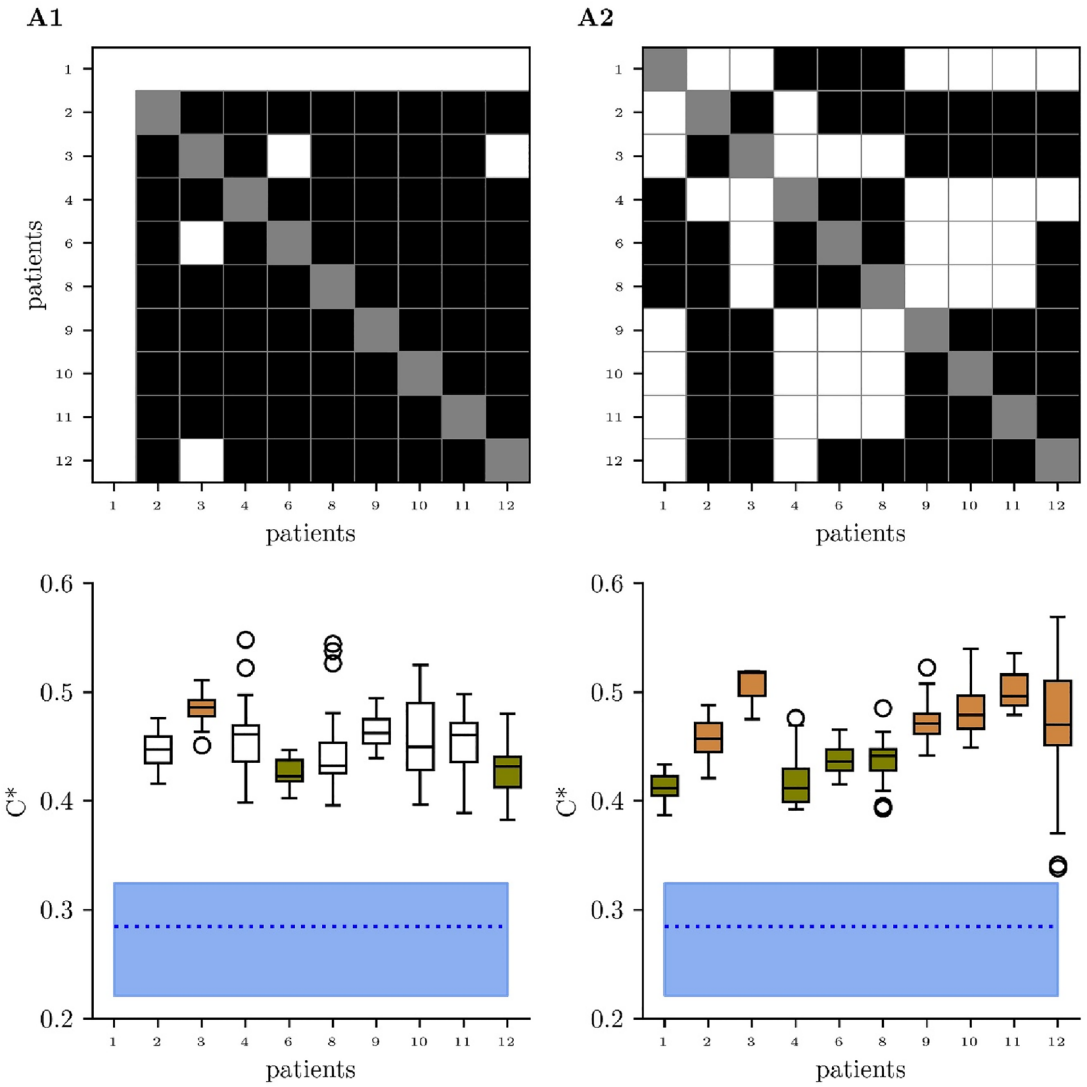
Results of between-patient comparisons (Scheffé tests; top) and distributions of age-corrected average clustering coefficients C* (bottom) at assessments (**A1**,**A2**). White rectangles indicate significant differences (*p* < 0.05). Regarding the distributions of C*, bottom and top of a box are the first and third quartiles, and the black band is the median of the distribution, respectively. The ends of the whiskers represent the interquartile range of the data. Outliers are marked by an o-sign. Blue dotted lines and shaded areas indicate median and range of age-corrected average clustering coefficients C* of healthy controls. At assessment (**A1**), C* of patient 3 (orange) differs significantly from the values of patients 6 and 12 (green). At (**A2**), C* of patients 1, 4, 6, and 8 (green) differ significantly from the values of patients 2, 3, 9, 10, 11, and 12 (orange). The EEG recording of patient 1 during B1 at A1 had to be excluded from the analyses because of too many artefacts.

As already mentioned above, at A1 all patients were clinically indistinguishable classified as UWS (“Methods”; CRS-R between 2 and 8). At A2 (3–6 weeks later), two patients were categorized as being improved from UWS to MCS (one patient with CRS-R score changing from 3 to 8, another with a CRS-R score changing from 6 to 14), while for the other eight patients the status UWS remained. Our findings obtained with age-corrected average clustering coefficients C* (Scheffé test) considerably contrast this clinical classification. At A1, three patients (3, 6, and 12) differed from at least one other patient. In particular, patient 3 with the highest C* value (more distant from the values of the healthy controls) differed from patients 6 and 12 with significantly lower C* values (closer to healthy controls). At A2, all 10 patients differed from at least two other patients (Fig. 4) and could be assigned to two distinct groups: group 1 included patients 2, 3, 9, 10, 11, and 12 with higher C* values (more distant from the values of the healthy controls). Group 2 included patients 1, 4, 6, and 8 with lower C* values (closer to healthy controls). Both these groups, however, did not differ in regard to clinical or demographic features (“Methods”).

### Global network characteristics index individual short-term and long-term treatment effects

**Figure 5:**
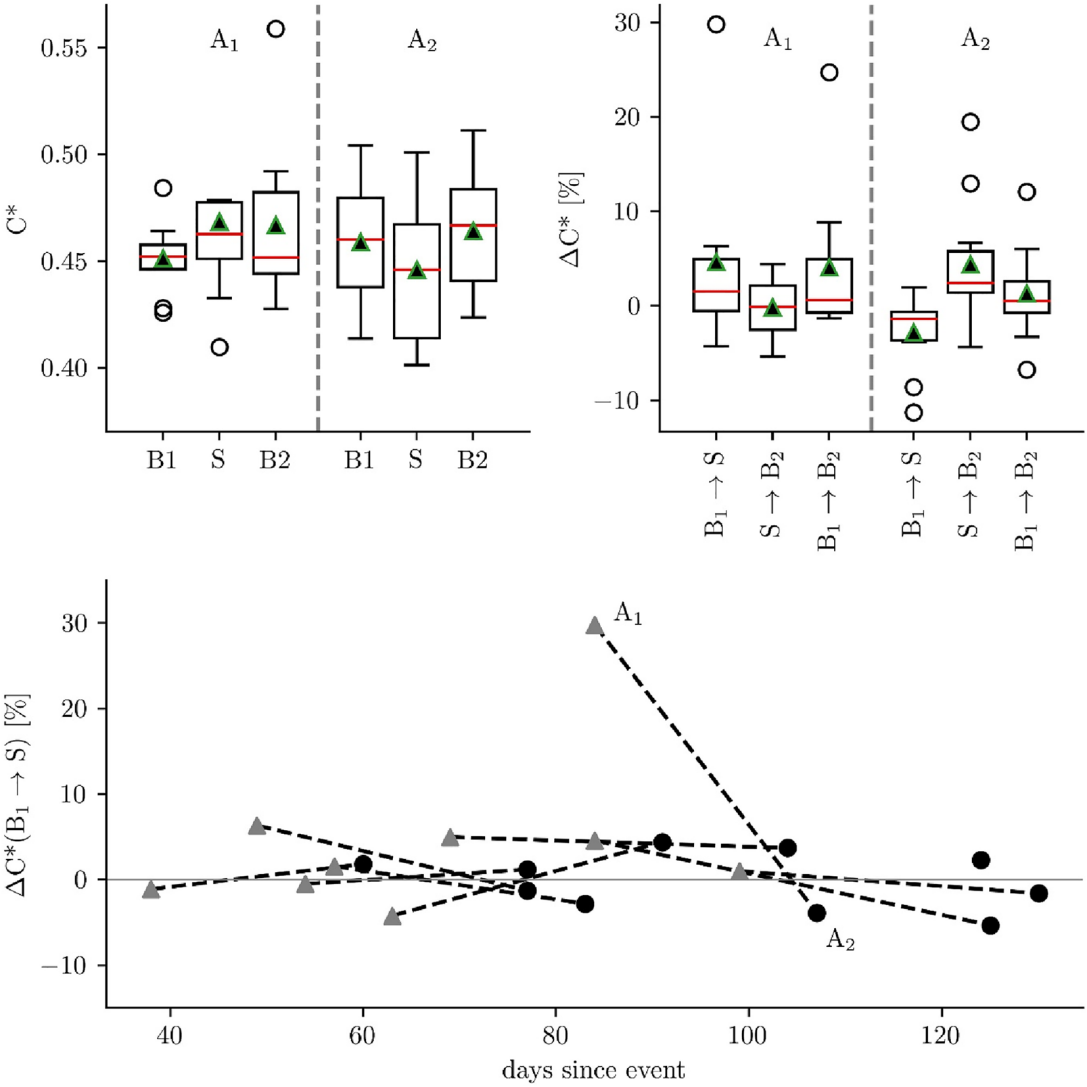
*Top:* Sample distributions of age-corrected average clustering coefficients C* of evolving functional brain networks during periods B1, S, and B2 (left) as well as of stimulation-induced changes ΔC* between periods (right) at first (**A1**) and second assessment (**A2**). Properties of boxplots as in Fig. 2. *Bottom:* Individual stimulation-induced changes ΔC* between periods B1 and S at first (**A1**: grey triangle) and second assessment (**A2**: black dot) on a time line starting with injury.

In a next step, we examined whether short-term and long-term treatment affects evolving functional brain networks. On the sample level (Fig. 5 top), we observed the age-corrected average clustering coefficient C* to exhibit—for each period of our experimental schedule (B1, S, B2) and at both assessments (A1, A2)—a large interindividual variability. The same applied for stimulation-induced changes of C* (ΔC*), and we could not observe any statistically significant modifications. Searching for the source of the large interindividual variability, we inspected individual stimulation-induced changes of C* from period B1 to period S (referred to as *short-term treatment effect*), thereby taking into account the individual time since injury (Fig. 5 bottom). Despite the large interindividual variability, there appeared to be an overall decreasing tendency between values of ΔC* assessed at A1 and at A2 (referred to as *long-term treatment effect* due to continued rehabilitation complex treatment) which became more pronounced with an increasing temporal distance to injury. In order to corroborate this observation, we performed statistical tests for each patient separately and report our findings in Tables 1 and 2. In about a third of patients (Table 1), we observed significant stimulation-induced changes of C* (both from baseline period B1 to the stimulation period S and back to the baseline period B2; short-term treatment effect) at A1 and A2. In addition, at A1 we observed for three patients a possible enduring effect of the stimulation, which appeared to be masked by a floor effect at A2. Of note, we observed significant changes of C* for period B1 in two patients between assessments A1 and A2, for period S in five patients and for period B2 in seven patients, possibly indicating a combined impact of the short-term stimulation (enduring effect) and the long-term treatment on evolving functional brain networks (Table 2).

**Table 1:** Number of patients with significant changes in age-corrected average clustering coefficient of evolving functional brain networks (*p* < 0.05) between periods (B1, S, B2) of the experimental schedule (range of Wilcoxon signed rank test *W* values in brackets).

Assessment	B1 → S	S → B2	B1 → B2
A 1	3 patients (0; 100)	2 patients (54; 103)	3 patients (2; 18)
A 2	3 patients (0: 86)	3 patients (1; 82)	1 patient (89)

**Table 2:** Number of patients with significant changes in age-corrected average clustering coefficient of evolving functional brain networks (*p* < 0.05) between assessments A1 and A2 for each period (B1, S, B2) (range of Wilcoxon signed rank test *W* values in brackets).

Assessment	B1	S	B2
A 1 → A 2	2 patients (17; 41)	5 patients (0; 19)	7 patients (3; 93)

**Figure 6:**
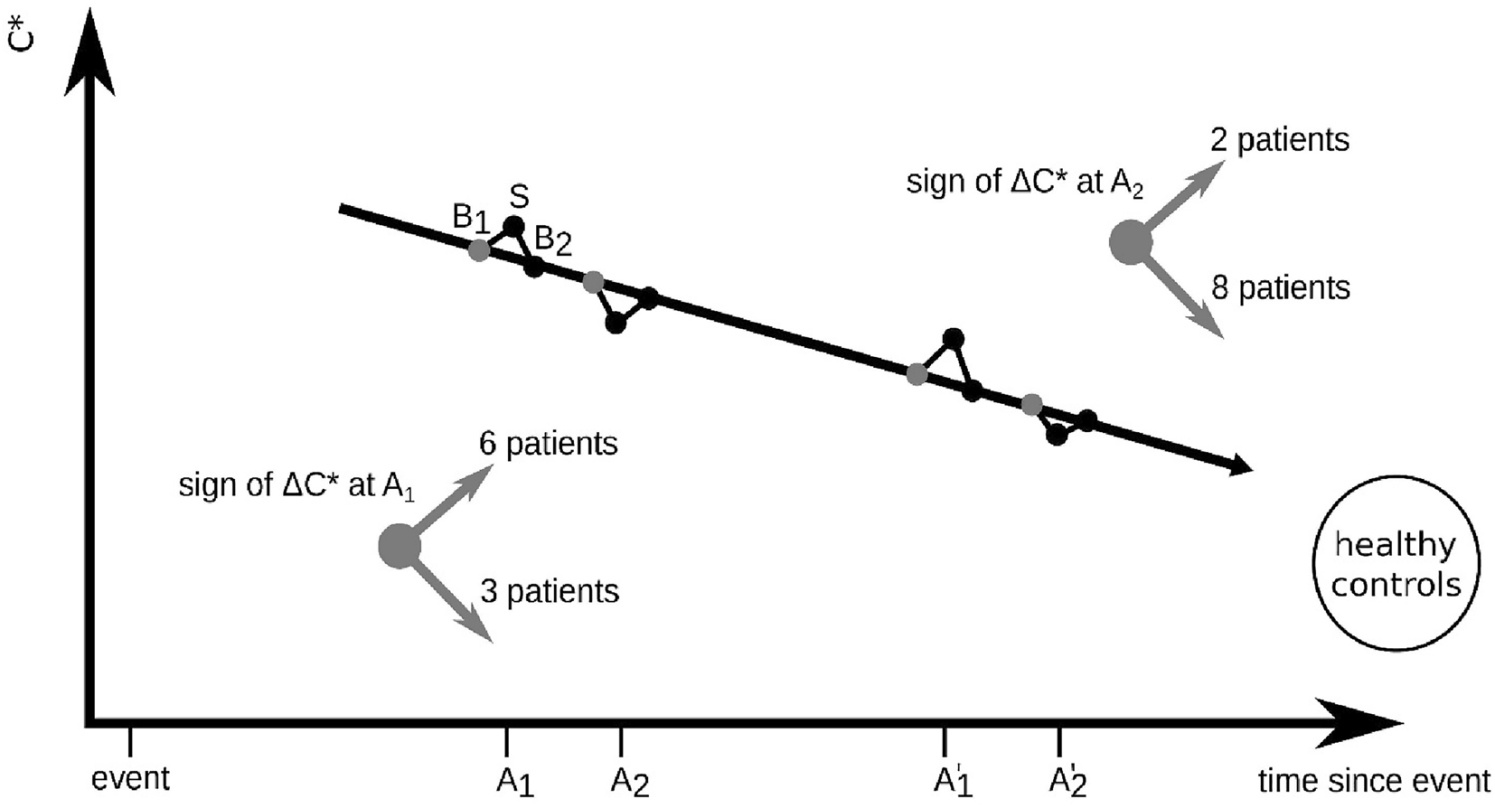
Proposed model of short-term and long-term stimulation-induced modifications of evolving functional brain networks in patients with an unresponsive wakefulness syndrome. The black arrow indicates a long-term treatment effect due to continued rehabilitation complex treatment as monitored with age-corrected average clustering coefficient C* at different assessments (**A1**,**A2**). Short-term modifications can be monitored with changes of C* (ΔC*) while evolving functional brain networks transit from a baseline period at rest (**B1**) to a period of sensory stimulation (**S**) and back to a baseline period at rest (**B2**). A negative change (ΔC* < 0 for B1 → S) is considered as favorable. It can be observed in an increasing number of patients at (**A2**), while negative changes decrease. The general tendency towards a more segregated network (decreasing C*), as seen for healthy controls, indicates success of therapy and thereby recovery from injury.

Taking into account the aforementioned tendency for C* to decrease with an increasing temporal distance to injury (Fig. 5 bottom), we investigated whether additional information about stimulation-induced network modifications can be obtained when considering the direction of the (not necessarily significant) change of C* upon stimulation (B1 → S). Indeed, at A1 we observed C* to decrease in three out of ten patients when their evolving functional brain networks transited from period B1 to period S. There was no change in one patient, and C* increased in six patients. Interestingly, at A2 we observed C* to decrease in eight out of ten patients, while C* continued to increase in two hypoxic patients who showed this reaction already at A1 (cf. Fig. 6).

## Discussion

Disorders of consciousness (DOC) and diagnostic differentiations within and between conditions of unresponsive and minimally responsive wakefulness (UWS/MCS) represent an increasing diagnostic and therapeutic challenge in early rehabilitation. There is now ample evidence that electrophysiological and neuroimaging techniques can provide valuable additional information to clinical routine^10, 15, 18, 21, 22, 24, 27^ However, these techniques with highly diverse assessments and complicated computational requirements are not always available in neuro-rehabilitation centers. Particularly fMRI is expensive and not the rule on-site. Current research activities are in an experimental state whilst standardized time- and personal-efficient solutions would be appreciated. EEG is still the most available tool. It is safe, comparably cheap, and it provides great opportunities for quantitative analyses which could be automated.

In the present study, we chose a practical and simple way for assessing and monitoring DOC in early rehabilitation by combining already used methods: repeated EEG recordings, basal multisensory stimulation, and clinical coma ratings. EEG data were recorded continuously following a block design (rest–stimulation–rest) during two routine examinations (3–6 weeks apart). In a time-resolved manner, evolving functional brain network analyses were carried out by estimating interdependencies between EEG time series from pairs of brain regions, regardless of their anatomical connectivity. In the literature, this type of analysis is dealt with under the term “complex network analysis” as compared to spectral, or connectivity, or complexity analyses^27^. To keep the analysis as simple as possible as well, two commonly used global characteristics of segregation and integration of evolving functional brain networks were assessed: average clustering coefficient and average shortest path length. We recently used a similar approach to monitor modifications of large-scale evolving functional brain network from subjects with epilepsy due to external brain stimulation and biofeedback^28, 29^. Aiming at an analysis at the individual level, ten patients with heterogeneous brain lesions were evaluated who—according to the clinical consensus and the results of the CRS-R—were all diagnosed with unresponsive wakefulness syndrome (UWS). In addition, in order to have a reference point for the interpretation of the patient data, one-time assessments of nine healthy subjects were performed.

Before answering the three research questions of our study, two methodological issues need to be dealt with briefly. Firstly, there was a considerable interrelation between the network characteristics average clustering coefficient and average shortest path length, which however could be expected, at least to some extent, given our network construction. It therefore appeared sufficient to base downstream analyses on the average clustering coefficient. Secondly, there was a considerable and reliable negative correlation between the average clustering coefficient and age, which was apparent at both assessments (A1 and A2) in patients, and in the one-time assessment of the healthy controls as well. An age-dependence of some characteristics of EEG-derived functional brain networks has been reported before^30, 31^. The strength of the age dependency made it necessary to correct the obtained data for age in order to avoid an overlap with other time dependencies (see below).

### Do global network characteristics allow to differentiate patients in clinically indiscriminate states of unresponsive wakefulness (UWS)?

Our first research question can be answered positively. Using age-corrected clustering coefficients, the results show low heterogeneity of patients at the first assessment A1 but definitively a higher one at the second assessment A2. First two, and later all patients could be differentiated from at least one other patient. As indicated by patient-to-patient comparisons, two significantly different groups could be discerned at A2, 6 patients with higher and four patients with lower scoring clustering coefficients. Whether such resting state differences will improve patient classification in UWS in the future needs to be determined. The same applies for stimulation and time associated changes which will be discussed in the next paragraphs.

### Do global network characteristics indicate functional recovery and success of continuous early rehabilitation complex treatment (long-term treatment effects)?

The increasing differentiation between patients over time—between assessments A1 and A2—may already be taken as first evidence for longitudinal modifications towards a less segregated network over time. At A2, two patients were clinically classified as MCS while the majority remained classified as UWS. Nevertheless, when taking into consideration intraindividual changes between A1 and A2, the age-corrected average clustering coefficients changed significantly in up to seven patients depending on the period of the experimental stimulation schedule. Despite the high interindividual variability, the direction of intraindividual changes over time and with an increasing temporal distance to injury tended towards the levels obtained for healthy control subjects, i.e. towards less segregated functional networks. We interpret this as improvement although it cannot be decided within the present study design as to whether this is due to therapy or due to the passage of time (spontaneous recovery).

### Do global network characteristics indicate the brain’s response to single basal multisensory therapeutic interventions (short-term treatment effects)?

Without a control condition—there is no ethical justification for not treating patients in early rehabilitation—it cannot be differentiated as to whether the observed positive development reflects spontaneous recovery, a positive impact of therapy, or both. In this regard the results provide quite an interesting picture: At A1, the age-corrected average clustering coefficient in response to the stimulation changed into a direction, which we interpret as less pathological in three patients. In one patient, there was no change, and in six patients it unexpectedly changed towards a more pathological direction. The situation was completely different at A2, where the age-corrected average clustering coefficients of eight patients changed into a favourable and of two patients into an unfavourable direction. These results indicate an increasing positive response to stimulation in the majority of patients. We speculate that the negative response may reflect an overstraining of the patients´ network due to sensory overload. Possibly such responses to stimulation can be used for controlling treatment selection and intensity of stimulations in the future. Interestingly, the two patients with unfavourable responses to stimulation at A2 (pts. 6 & 8) already had such responses at A1. These patients belonged to the post-hypoxic group, which was supposed to be more severely affected compared to the purely “lesional” group.

Summarizing our findings, we postulate the following model (Fig. 6): A decreasing age-corrected average clustering coefficient C*, assessed at different points in time, reflects the short-term stimulation-induced and the long-term continuous therapeutic intervention-induced modification of evolving functional brain networks towards a less segregated network. This is further corroborated by the observation that the time-dependent decrease of C* approached the averaged values seen for healthy controls, which we interpret as another indication for success of therapy and thereby recovery from injury. Overall, the results of our proof-of-principle study encourage the implementation of repeated analyses of resting- and stimulation-associated large-scale functional brain networks by merging what is already at hand and being done either way during early rehabilitation. The method, should it receive further confirmation in larger populations of patients not only with UWS but also with MCS, can easily be adapted by other centers. Surely, long-term follow-ups with reference to the final rehabilitation outcomes will be needed. Even automated quantitative EEG analyses appear possible. Artefact control, however, requires EEG expertise, and this represents a challenge regarding staff/personal resources and time. Future work may also focus on other, e.g. more local network characteristics and address questions of whether network characteristics provide information for the selection and control of individual therapies^32, 33^. At the present state, we refrained from more detailed analyses of the patients’ responses to different types of stimulation. For the purpose of this study, it appeared sufficient to merge all conditions into one multimodal stimulation condition. With more data also from partially conscious patients, separation of stimulation conditions might reveal additional valuable information for the monitoring and therapy of DOC. With a broader range of states of consciousness under study, a comparison of network reorganizations seen here with the ones reported for healthy subjects during different sleep stages^34, 35^ might help to gain further insight into mechanisms of how brains regain consciousness and recover from DOC respectively.

## Methods

### Patients

Table 3 summarizes the demographic and clinical data of the ten investigated patients. In three patients, the primary lesion was a stroke/hemorrhage (pts. 2, 4, and 9), three patients had a traumatic brain injury (TBI) (pt. 1), two of them with hemorrhage (pts. 2 and 6) and one patient with TBI experienced hypoxia after cardiopulmonary resuscitation (pt. 6) as did two other patients (pts. 11, and 12).

**Table 3:** Patient characteristics.

Patient ID/gender	Age	Lesion	1. assessment (A1)			2. assessment (A2)		
			Days since injury	CRS-R points	DOC	Days interval	CRS-R points	DOC
1/m	78	Bifrontal TBI & ICB right ACM	98	6	UWS	26	14	MCS
2/ m	74	TBI right thalamic hemorrhage ventricular extension	99	7	UWS	31	5	UWS
3/ f	80	ICB Ventricular extension	38	3	UWS	22	8	MCS
4/ m	54	Cerebral infarct right and left MCA & left VA	84	4	UWS	41	4	UWS
6/ f	72	TBI, SDH & hypoxia after CA	49	4	UWS	28	5	UWS
8/ m	52	SAB & hypoxia after CA	84	5	UWS	23	5	UWS
9/ m	69	Hemorrhage basal ganglia right & ventricular extension	54	4	UWS	23	4	UWS
10 / m	40	Cardiac infarct,, hypoxia, global brain edema	57	8	UWS	26	4	UWS
11 / f	53	Hypoxia after CA	69	3	UWS	35	2	UWS
12 / m	48	Hypoxia after CA	63	2	UWS	28	5	UWS

*CRS-R* coma recovery scale revised, *DOC* disorder of consciousness, *UWS* unresponsive wakefulness syndrome, *MCS* minimally conscious syndrome, *CA* cardiac arrest, *ICB* intracerebral bleeding, *SDH* subdural hemorrhage, *SAB* subarachnoid bleeding, *TBI* traumatic brain injury, *MCA* medial cerebral artery, *VA* vertebral artery.

(Two patients (with IDs 5 and 7) were excluded because they had no follow-up assessment).

Potential CNS active drugs which may have affected EEG or cognition were antiseizure drugs (levetiracetam, carbamazepine, valproate, or pregabalin) in all but one patient (pt. 12) at both assessments. One patient (pt. 11) was additionally on pain medication at both assessments. No patient received barbiturates, benzodiazepines, or narcotics.

### Behavioral assessment—CRS-R

For the behavioral assessment of consciousness, the revised Coma Recovery Scale (CRS-R) is being applied in addition to clinical consensus as part of the clinical routine by trained staff, which is familiar with the CRS-R scoring system^36, 37^. The CRS-R is an internationally accepted instrument for the monitoring of disorders of consciousness providing a rough estimate of the functional outcome as well^38^. The CRS-R encompasses six subscales that are each built in a hierarchical order with increasing scores indicating an increase in complexity of behavior. The subscales are categorized by responses in oromotor, motor, visual, auditory, communication and arousal functions. The sum of subscales, the total CRS-R score, ranges between 0 and 23. Low scores reflect reflexive behavior, higher scores more cognitive behavior.

In order to limit statistical complexity in the small group of patients, only the total CRS-R score was chosen as a measure for every patient and rated as UWS and MCS. Published CRS-R cut off scores for a differentiation between UWS from MCS range between 8 and 10^39, 40^. Taking this into consideration, we set the cut off to 8 points.

### Continued complex rehabilitation treatment

All patients underwent standardized “early rehabilitation complex treatment” (according to the guidelines of the German DRG, OPS 8–552). The procedure provides continuous involvement of neurological or neurosurgical expertise, and the following therapeutic areas are available: physiotherapy, occupational therapy, neuropsychology, speech therapy / facial-oral therapy. Therapeutic interventions were carried out on a daily base in different combinations of at least 300 min´ treatment duration (with simultaneous involvement of two or more employees).

### Neurophysiological assessment—EEG

A 19-channel EEG system (Nihon Kohden Europe GmbH) was used, and electrodes were placed at locations FP2, FP1, F8, F7, T8, T7, P8, P7, F4, F3, C4, C3, P4, P3, Fz, Cz, and Pz with an additional reference electrode on the forehead. EEG data were sampled at 200 Hz using a 16 bit analogue-to-digital converter. Data were band-pass filtered offline between 1–45 Hz (4th order Butterworth characteristic), and additionally, we used a notch filter (3rd order) to suppress contributions at the line frequency (50 Hz). We visually inspected all recordings for strong artefacts such as subject movements, amplifier saturation, or stimulation artefacts. For the following analyses, such data were excluded due to too many artefacts.

### Constructing and characterizing evolving functional brain networks

To construct a time-dependent sequence of weighted and fully connected functional brain networks from the 30 min EEG recordings, we associated network nodes with EEG electrodes and inferred network links by estimating—in a time-resolved manner—interdependencies between EEG time series from pairs of brain regions, regardless of their anatomical connectivity^41–44^. To this end, we calculated a synchronisation index *r*_*ij*_ between time series of instantaneous phases from all pairs of brain regions (*i,j*) sampled by the EEG electrodes with a sliding window approach^45, 46^. Non-overlapping windows had a duration of 10 s (2000 data points). The synchronisation index is confined to the unit interval: *r*_*ij*_ = 1 indicates fully phase-synchronised brain regions and *r*_*ij*_ = 0 indexes an absence of phase synchronisation.

An important property of this estimation approach is that the instantaneous frequency (particularly in case of two or more superimposed oscillatory components) relates to the predominant frequency in the Fourier spectrum^47^. This predominant frequency may be subject to fluctuations in the EEG time series, and consequently the instantaneous frequency can vary rhythmically around the predominant frequency resulting in spurious estimates of the instantaneous phase. Such effects can be reduced, e.g., by taking the temporal average^45, 46^. From an electrophysiological point of view, however, it might be more reasonable to look adaptively (e.g., via the Hilbert transform as done here) at interdependences between predominant rhythms in the EEG than to look at interdependences in some a priori fixed frequency bands for which there is no power in the time series^46^.

In order to characterise a network’s global topological properties, we estimated its average clustering coefficient and its average shortest path length. The clustering coefficient is a measure of the degree to which nodes in a network tend to create cliques of strongly interconnected nodes. We calculated the average clustering coefficient as the mean of clustering coefficients computed for all nodes, where we employed the definition of the clustering coefficient for weighted networks^48^. The average clustering coefficient characterises the network’s functional segregation; the higher its value, the more segregated is the network.

The average shortest path length is defined as the average number of steps along the shortest paths for all possible pairs of network nodes. For our weighted networks, we defined the ‘length’ of a path between a pair of nodes as the inverse of the weight of the link along the path connecting the nodes^49^. We computed the average shortest path length using an algorithm proposed by Dijkstra in 1959^50^ to characterise the network’s functional integration; the shorter the length, the more integrated is the network.

*C* and *L* represent the mean values of average clustering coefficients and average shortest path lengths of the snapshot networks in a given period (B1, S, B2) of our experimental schedule. On average, the number of snapshot networks in period B1 amounted to 31, in period S 51, and in period B2 33.

### Statistical analyses

In order to test for stimulation-induced changes of network characteristics (within the experimental schedule and between the two assessments, i.e., within-patient comparisons), we performed nonparametric Mann–Whitney U tests for independent variables and Wilcoxon signed rank tests for dependent variables. We considered differences significant at *p* < 0.05 (after Bonferroni correction, whenever appropriate). Differences between network characteristics from individual patients (i.e., between-patient comparisons) were assessed using post hoc Scheffé tests (*p* < 0.05).

### Ethics statement

The study protocol had been approved by the ethics committee of the University of Düsseldorf (ID 2017114499) before the study has started. All experiments were performed in accordance with relevant guidelines and regulations.

### Informed consent statement

Informed consent was obtained from the legal guardian(s) of all included patients.

## Data Availability

The data that support the findings of this study are available from the corresponding author upon reasonable request. The data are not publicly available as they contain information that could compromise the privacy of research participants.
